# Biological evaluation of critical bone defect regeneration using hydroxyapatite/ alginate composite granules

**DOI:** 10.1590/acb392824

**Published:** 2024-07-22

**Authors:** Iorrana Índira dos Anjos Ribeiro, Renata dos Santos Almeida, Ana Maria Guerreiro Braga da Silva, Aryon de Almeida Barbosa, Alexandre Malta Rossi, Fúlvio Borges Miguel, Fabiana Paim Rosa

**Affiliations:** 1 Universidade Federal da Bahia – Instituto de Ciências da Saúde – Salvador (BA), Brazil.; 2 Universidade Federal do Recôncavo da Bahia – Centro de Ciências Agrárias, Ambientais e Biológicas – Cruz das Almas (BA), Brazil.; 3 Centro Brasileiro de Pesquisas Físicas – Departamento de Física Aplicada – Rio de Janeiro (RJ), Brazil.; 4 Instituto de Patologia Cutânea – Salvador (BA), Brazil.

**Keywords:** Alginate, Biocompatible Materials, Rats, Durapatite, Bone Regeneration

## Abstract

**Purpose:**

to evaluate biocompatibility and osteogenic potential of hydroxyapatite/alginate composite after its implantation on rat calvarian critical bone defect.

**Methods:**

thirty adults male Wistar rats were randomly distributed into two groups: GHA – critical bone defect filled with hydroxyapatite/alginate composite granules (HA/Alg) and CG – critical bone defect without biomaterial; evaluated at biological points of 15, 45 and 120 days.

**Results:**

the histomorphometrically analyses for GHA showed osteoid matrix deposition (OM) among the granules and towards the center of the defect in centripetal direction throughout the study, with evident new bone formation at 120 days, resulting in filling 4/5 of the initial bone defect. For CG, this finding was restricted to the edges of the bone margins and formation of connective tissue on the residual area was found in all biological points. Inflammatory response on GHA was chronic granulomatous type, discrete and regressive for all biological points. Throughout the study, the CG presented mononuclear inflammatory infiltrate diffuse and regressive. Histomorphometry analyses showed that OM percentage was evident for GHA group when compared to CG group in all analyzed periods (p > 0.05).

**Conclusions:**

the biomaterial evaluated at this study showed to be biocompatible, bioactive, osteoconductive and biodegradable synchronously with bone formation.

## Introduction

Use of different materials to replace lost tissue is documented since antiquity, with records dated since the Ancient Egypt. However, scientific and technological advances of the last few decades made it possible exponential development and use of biomaterials. Although the osseous tissue is dynamic and has an excellent regenerative and remodeled capacity, when it comes to great tissue loss, the regenerative capacity becomes limited and its repair usually is made with fibrous connective tissue^1^^-^ 4. Many of these tissue losses affects function and aesthetics which, consequently, interferers on wellbeing of the affected individuals. Therefore, given these limitations, different types of biomaterials have been developed and improved aiming to stimulate or promote bone regeneration.

There are several option of biomaterials, including metallics, ceramics, polymeric or composite, which are produced in different forms and presentation formats, such as gel, powder, sponges, tablets, cylinders, microspheres, membranes, three-dimensional matrices, and granules. Composite biomaterials synthesized from combined different materials, which have different phases or classes, have an important role in research regarding tissue bioengineering area. This association minimizes limitations and enhances the physical-chemical properties of each material used, as well as make it possible to mimic some tissues, according to its applicability. For bone regeneration specifically, use of ceramic and polymer composites have been increasing frequently due to improvement in mechanical properties and biological performance, as these materials seem to be similar to bone tissue composition as a result of their organic and inorganic proportions.

Thus calcium phosphates stand out, amongst which are hydroxyapatite (HA), a ceramic with good biocompatibility, osteoconduction, osteointegration, thermodynamic stability at physiological pH and chemical similarity to biological apatite^5^^,^ 6, and alginate (Alg), a biopolymer extracted from seaweed, hydrophilic, non-toxic, non-immunogenic, biocompatible, biodegradable^7^^,^ 8, and bioabsorbable. Combined they allow the formation of pores on the structure of the material when in contact with tissue fluids in vivo^9^.

The physicochemical properties of each biomaterial directly interfere with tissue response after in vivo implantation. Thus, before their clinical use, it is crucial to carry out studies that evaluate biological behavior to guarantee the efficacy and safety of these materials. Therefore, the aim of this study was to evaluate the biocompatibility and osteogenic potential of hydroxyapatite and alginate (HA/Alg) composite after implantation on critical bone defect in rat calvaria.

## Methods

### Animals and ethical considerations

Experimental procedures of this study were carried out at the Institute of Health Sciences (ICS), Federal University of Bahia (UFBA), in accordance with the Ethical Norms for Research on Animals (Law No. 11.794 of 2008), after approval by the Ethics Committee on Animal Use (CEUA) of this institution. Thirty adults male Wistar rats, weighing between 350 and 450g, three to four months old were randomly assigned to two experimental groups: GHA – critical bone defect filled with HA/alg composite granules and CG (control group) – empty critical bone defect. These animals were evaluated at biological points of 15, 45 and 120 days postoperative, with five animals in each group/period. Throughout the study, animals were kept in proper propylene boxes, bedded with autoclaved wood shavings and identified according to group and biological point. During the experiment, animals received rat food and water *ad libitum*, in a fountain appropriate for rats.

### Synthesis and processing of HA/Alg composite

The biomaterials evaluated in this study was synthesized, processed, characterized, sterilized and supplied by Biomaterials Laboratory (LABIOMAT) researchers from Brazilian Center for Physical Research (CBPF), Rio de Janeiro, Brazil.

HA synthesis was carried out by a mixture of a dibasic ammonium phosphate solution, kept at a pH greater than 12, to a solution of calcium nitrate [Ca(NO_3_)^2^], compound which remained under constant agitation. The formed precipitate was filtered, washed, and then added to 1% sodium alginate solution, in a ratio of 15:1, under slight agitation until a homogeneous paste was obtained. Solids were oven dried at 80 °C for 24h, macerated to form granules and separated using sieve to select granules with an aperture between 425 and 600μm mesh. These granulates were separated into equal parts and aliquoted in Eppendorf tubes for sterilization by gamma rays at 15 kGy/sample for 760 minutes.

Physical-chemical characterization of the HA/alg composite Superficial area. This characterization was obtained by BET (Brunauer, Emmett and Teller) method (ASAP 2020, Micromeritics Instrument Corporation^®^, Norcross, Georgia, United States of America), which showed that HA evaluated in this study had a 35.95 m^2^/g superficial area.

Chemical analyses. Through Atomic Absorption Spectrometry (AAS) (Shimadzu AA 6800, Shimadzu Corporation^®^, Chiyoda, Tokio, Japan) it was possible to identify molar calcium/phosphate ratio (Ca/P) of approximately 1.67, and chemical composition of the material, as showed in Table 1.

**Table 1:** AAS of the HA/Alg composite.

Sample	% Ca	mol of Ca	% P	mol of P	Ca/P
HA	35.70	0.8908	16.40	0.52948	1.6823
HA	36.00	0.8982	16.60	0.53593	1.6760
HA	37.12	0.9262	17.20	0.55530	1.6679
Mean value					**1.6754**

**Figure 1:**
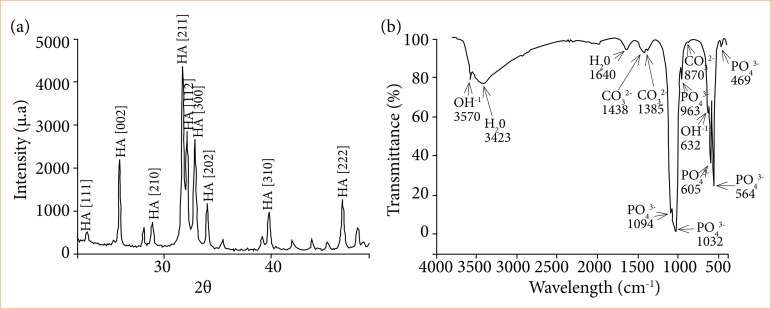
XRD and FTIR analyses from the HA/Alg composite. **(a)** Peaks are noted due to HA crystallinity pattern, in accordance with the standard sheet PCPDFWIN 09.0432. **(b)** Intense and large water bands are observed at 3430 and 1646 cm^-1^ regions. On 1462 to 1414 cm^-1^ region, carbonate ions characteristic bands were found. On 1038, 961, 602 and 560 cm^-1^ regions, phosphate ions characteristic bands were found. Hydroxyl ions bands were observed at 3570 and 635 cm^-1^ regions.

X-ray diffraction (XRD). Microspheres crystallinity was determined by XRD technique, using high-resolution diffractometer (HZG4, Zeiss^®^, Jena, Thuringia, Germany) operating at 30 kV and 15 mA with CuKα radiation (λ= 1.542Å), at standard sheet PCPDFWIN 09.0432 (Joint Committee on Powder Diffraction Standards – JCPDS). For these analyses, diffractogram showed peaks corresponding to the crystalline profile of a standard HA (Fig. 1a).

Fourier Transform Infrared Spectroscopy (FTIR). FTIR (Schimadzu IR-Prestige 21, Shimadzu Corporation^®^, Chiyoda, Tokio, Japan) analyses revealed typical HA bands for phosphates (PO_4_^3-^), water (H_2_O) e hydroxyl (OH^-^). On regions 1462 and 1414 cm^-1^ carbonate ions characteristic bands were noticed, due to the Alg used to synthesize this composite. Furthermore, water bands proved that microspheres were not heat treated (Fig. 1b).

### Surgical procedures

**Figure 2:**
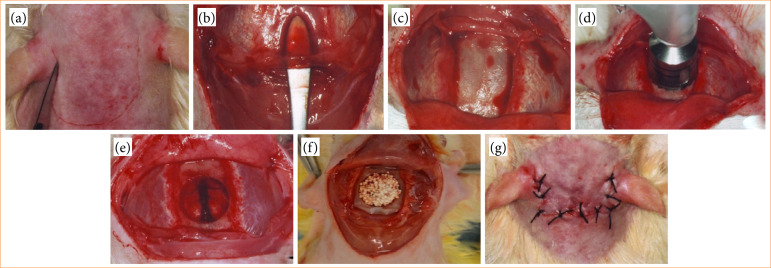
Surgical steps to create critical bone defect and implantation of biomaterial. **(a)** bicoronal semilunar skin incision; **(b)** detachment and removal of periosteum; **(c)** exposed osseous tissue; **(d)** critical bone defect made by trephine drill; **(e)** critical bone defect; **(f)** implantation of biomaterial; and **(g)** skin tissue flap repositioned and sutured in simple interrupted pattern.

Prior to surgical procedure, animals received anesthesia and analgesia by intraperitoneal injection of ketamine hydrochloride (100 mg/kg) and xylazine hydrochloride (10 mg/Kg), respectively. Animals were placed in prone position, trichotomized and submitted to surgical antisepsis with 1% alcoholic chlorhexidine at calvaria region. A three-centimeter long bicoronal semilunar skin incision was made with a 15-scalpel blade (Fig. 2a), and blunt divulsion of soft tissues was done with blunt tip scissors. Subsequently, periosteum was removed (Fig. 2b) to expose the osseous tissue (Fig. 2c) and to create the critical bone defect (Fig. 2d e 2e) between the vertices of the anterior and posterior calvarial sutures. Then, bone defects were created using trephine drill with a contra angle diameter of 8mm with 16:1 reduction coupled to an implant motor at 1500 rpm, under constant irrigation with saline solution. After the creation of the bone defect, biomaterial was implanted (Fig. 2f), according to the experimental group, except for the CG group, in which the bone defect remained without biomaterial implantation. Finally, the skin tissue flap was repositioned and sutured with 4.0 silk thread in a simple interrupted pattern (Fig. 2g).

### Laboratory stage and histomorphometric analysis

Animals were euthanized 15, 45 and 120 days postoperative with intraperitoneal lethal dose of ketamine hydrochloride (300 mg/kg) associated with xylazine hydrochloride (30 mg/Kg). The biological points were defined following previous research by this research group^1^^-^ 4 ^,^ 10 ^,^ 11. The upper portion of calvaria bone was removed, discarding all surrounding soft tissue, and fixed in 4% formaldehyde for 72 hours. The next step involved reducing the anterior, posterior, and lateral portions of the calvaria bone, followed by a decalcification process in a 7% nitric acid solution for approximately two hours. Specimens were embedded in paraffin, blocks were sectioned at 5μm thickness in transversal direction of bone defect, and stained with hematoxylin-eosin (HE), picrossírius red (PIFG) and Masson-Goldner’s trichrome (TG).

All histologic cuts were analyzed by common light microscopy and recorded by DFC310FX digital camera (Leica^®^, Wetzlar, Germany) coupled to DM6 B optic microscope (Leica^®^, Wetzlar, Germany). LAS software – Leica Application Suite (Leica^®^, Nußloch, Baden-Württemberg, Germany) was used for the histomorphometric analysis to measure newly formed osteoid matrix percentage (% OM) compared to total defect area.

### Statistical analysis

All data were described as mean value and standard deviation. To compare differences between groups, a non-parametric Wilcoxon signed rank test and analysis of variance (ANOVA), with software *R* ^®^, was done using a 5 % level of significance (p < 0.05).

## Results

### Histomorphological analysis

**Figure 3:**
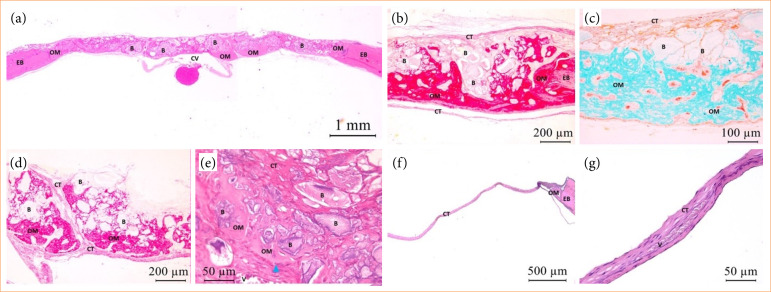
GHA and CG photomicrographs at 15 days postoperative. OM with osteoblasts (blue triangle) adjacent to EB (GHA and CG) and central region of the defect, within the biomaterial **(B)** (GHA). In both experimental groups, CT formation was noted in the residual area of the defect, with the presence of blood vessels **(V)**. Central vein (CV). HE staining – **(a)** HE (2.5x); **(b)** PIFG (10x); **(c)** TG (20x); **(d)** PIFG (10x); **(e)** HE (40x); **(f)** HE (5x); **(g)** HE (40x).

Bone neoformation near the edges of bone defect (EB) with active osteoblast was noted in both groups, at biological point of 15 days postoperative, evident in GHA. For this group, osteoid matrix (OM) formation towards the defect in centripetal direction was seen between and in direct interface with biomaterial particles, mainly in the region closest to the dura mater. The remaining area was notably filled by biomaterial and neoformed connective tissue (CT) throughout its entire length and around 2/3 of the defect’s thickness. For CG, mineralized tissue formation remained restricted to EB and a residual area was filled with well-vascularized CT. Both groups presented mildly chronic type inflammation. However, in addition to mononuclear inflammatory infiltrate, some multinucleated giant cells were observed mostly surrounding the biomaterial granules for GHA, characterizing this inflammatory response as chronic granulomatous type (Fig. 3).

**Figure 4:**
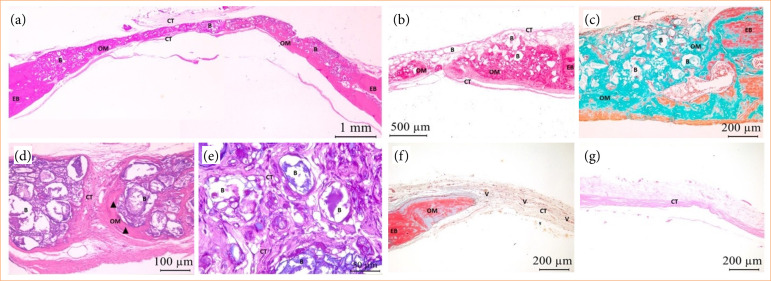
GHA and CG photomicrographs at biological point of 45 days. Notice OM adjacent to EB and in centripetal direction, within biomaterial **(B)**, with osteocytes (black arrow). CT with large amount of blood vessels **(V)**. **(a)** HE (2.5x); **(b)** PIFG (5x); **(c)** TG (10x); **(d)** HE (20x); **(e)** HE (40x); **(f)** TG (10x); **(g)** HE (10x).

At 45 days, bone growth in both groups reached a similar level as it did at the 15-day biological point. This finding was seen organized and inside the biomaterial granulates, with osteocytes present, for GHA. Residual area was fulfilled by biomaterial, which was fragmented, and by vascularized CT. The formed CT at the remaining area exhibited a thinner thickness and denser appearance for CG when compared to what was observed in the previous period. At this biological point, chronic inflammation was scarce for both groups (Fig. 4).

**Figure 5:**
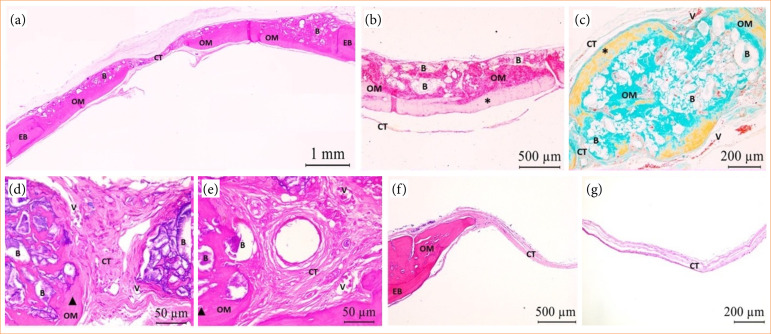
GHA and CG photomicrographs at biological point of 120 days. Notice OM deposition associated to EB in centripetal direction surrounding biomaterial **(B)**, with osteocytes (black arrow) and concentric lamellas (*). Dense and vascularized CT **(V)**. **(a)** HE (2.5x); **(b)** PIFG (5x); **(c)** TG (10x); **(d)** HE (40x); **(e)** HE (40x); **(f)** HE (5x); **(g)** HE (10x).

At 120 days both groups presented bone neoformation associated with the defect’s EB. The OM deposition was more pronounced than in previous biological points, in both extension and thickness, for GHA. The matrix displayed a mature and organized appearance, often accompanied by concentric lamellae. When compared to other biological points, the biomaterial exhibited fragmented and less evident appearance along the defect with dispersed distribution of particles among newly formed OM. At the remaining area, CT was denser and well vascularized. CG showed that OM deposition remained restricted to EB with dense CT formation thinner than EB, when compared to what was observed for 15 and 45 days biological points. At this biological point, chronic inflammation with rare mononuclear inflammatory cells was scarce for both groups (Fig. 5).

### Histomorphometric analysis

The OM neoformation percentage was evident for GHA when compared to CG group, except at 15 days biological point. Comparison between GHA showed evident bone neoformation at 120 days when compared to other biological points (Table 2).

**Table 2:** GHA and CG neoformed osteoid matrix percentagem at 15, 45 and 120 days, mean value (±DP).

Groups	15 days	45 days	120 days	ANOVA
GHA	7.9 ± 6.1%	24.5 ± 18.0%	40.6 ± 12.3%	p = 0.001 S
CG	11.7 ± 9.0%	21.0 ± 18.0٪	25.4 ± 13.0٪	p = 0.272 NS
**Wilcoxon**	*p* = 0.125 NS	*p* = 0.999 NS	*p* = 0.0625 NS	
**ANOVA** (GHA x CG)	p = 0.399 NS			

## Discussion

In an attempt to overcome bone tissue regenerative limitations due to extensive losses, several types of biomaterials have been developed and improved to stimulate and enable bone regeneration to reestablish tissue structure and function. Hence, composite materials, mainly ceramics and polymer-based, gained prominence in biomedical research and applications due to their similarity to bone tissue composition. Therefore, this study evaluated a composite of HA and Alg on critical bone defect.

Bone defect causes tissue injury, vascular rupture and triggers cytokines release that initiate acute inflammation, which is replaced, approximately after 24 to 48 hours, by mononuclear inflammatory cells that characterize chronic inflammatory response^12^^-^ 15. Our results are consistent with what was previously described for both groups under evaluation. However, the biomaterial implanted group had a chronic granulomatous type of tissue response, due to granules, re perceived by the organism as foreign bodies^10^^-^ 13. These findings corroborate with results demonstrated by some studies^1^^-^ 4 ^,^ 10 ^,^ 11, which used different biomaterials for bone regeneration. Throughout our study, the inflammatory response was discrete and regressive, resulting in scarce inflammatory cells at the biological endpoint, highlighting biocompatibility of the evaluated composite.

A three-dimensional (3D) scaffold was formed on the defect area by adequate implantation and distribution of biomaterial, enabling cell adhesion, proliferation, and differentiation, as well as bone neoformation (40.6%)^1^. On the other hand, CG presented bone neoformation restricted to bone edges (25.4%) and the residual area was filled with connective tissue. Scaffold were fundamental for cellular events observed during repair. Limited formation of bone tissue in CG was associated with reduced arrival of growth factors and osteogenic cells on the central region of the defect. Histomorphological pattern found for this group is compatible with critical bone defect, which, according to Schimdzt and Hollinger^16^, does not regenerate throughout animal’s life and has been extensively reported by different studies evaluating biomaterials for bone regeneration^2^^-^ 4 ^,^ 10 ^,^ 11 ^,^ 17 ^,^ 18. This type of defect in rat calvaria is a widely accepted model for studies in bone tissue bioengineering, as it allows verifying the biocompatibility and effect of biomaterials on bone neoformation^1^^,^ 11 ^,^ 19.

All biological points presented OM at the edges and throughout the entire extent of the defect, between and in direct interface with biomaterial granules. They were statistically significant (p = 0.001) throughout the analyzed periods, which proved the osteoconductive potential presented by the HA/Alg composite. Osteoconduction is an essential characteristic of osteogenic biomaterials, as it enables adhesion and migration of cells towards the central region of the defect, with consequent vascular and bone neoformation^20^. Histological results obtained in our study are in accordance with Liang et al.^21^, which implanted HA/Alg-based scaffolds on non-critical bone defect in calvaria of beagles. However, in our study, the percentage of newly formed mineralized tissue was higher when compared to their study, 40.6% at 120 days and approximately 20% at 90 days, respectively. HA/Alg composite granules exhibited excellent osteogenic potential considering the size of the defect used in our study.

When in contact with biological fluids in vivo, Alg forms a gel that promotes gradual exchange of calcium ions for sodium on interstitial area and, consequently, degrades, is reabsorbed, and supports new tissue formation^7^^,^ 8 ^,^ 22. Hydrogel, biological fluid, and inorganic part of biomaterial interaction favored both bone neoformation^21^^,^ 23 and biomaterial degradation, evidenced by HA/Alg size reduction, especially at the end of the study. Similar results were described by Paula et al.^17^ for the same biological points in regard to biodegradation and new bone formation.

Alg association with HA mimics organic and inorganic phases of bone tissue and increases composite bioactivity, which probably contributed to evident bone neoformation presented by GHA when compared to CG. Cuozzo et al.^24^ observed that in the group in which HA/Alg microspheres were implanted in the critical bone defect, new bone formation was restricted to OB, which contrast with our findings. The difference between these results may be related to biomaterial format since granules were used at the present study. The format associated with particles spatial distribution on bone defect allowed appropriate interstitium formation and adequate surface area for cellular events to occur during bone repair. It allowed specifically OM deposition at the edges and on the central region of the defect.

Research with HA/Alg composite is imperative, given great results found in this study, and other formats production and testing, such as 3D scaffolds and different experimental models.

## Conclusion

Hydroxyapatite/alginate (HA/alg) composite granules evaluated at this study showed to be biocompatible, bioactive, osteoconductive and with slow and harmonious biodegradation with bone neoformation. Such characteristics make it promising for future clinical use.

## Data Availability

All data sets were generated or analyzed in the current study.
